# PI3K/Akt pathway and neuroinflammation in sepsis-associated encephalopathy

**DOI:** 10.1515/med-2025-1248

**Published:** 2025-08-07

**Authors:** Yang Guo, Yonghao Yu

**Affiliations:** Department of Anesthesiology, Tianjin Medical University General Hospital, Tianjin, China

**Keywords:** sepsis-associated encephalopathy, PI3K/Akt pathway, neuroinflammation, blood–brain barrier, cognitive dysfunction

## Abstract

**Background:**

Sepsis-associated encephalopathy (SAE) is a complex neurological complication of sepsis involving activation of microglia in the central nervous system (CNS), blood–brain barrier (BBB) dysfunction, neurotransmitter dysfunction, impaired brain metabolism, and mitochondrial dysfunction. Neuroinflammation is a critical component of the pathogenesis. The phosphatidylinositol 3-kinase/protein kinase B (PI3K/Akt) signaling pathway, as a key intracellular signaling pathway, plays a crucial role in regulating neuroinflammation, maintaining the integrity of the BBB, and promoting neuronal cell survival.

**Objective:**

This review aims to summarize the role of the PI3K/Akt pathway in SAE-associated neuroinflammation and highlights potential therapeutic targets and strategies for its management.

**Methods:**

We systematically reviewed recent basic and clinical studies on PI3K/Akt signaling pathway in neuroinflammation associated with SAE, as well as the development of pathway-specific agonists and inhibitors.

**Results:**

The PI3K/Akt pathway serves as a crucial intracellular signaling axis involved in the regulation of neuroinflammatory processes. Accumulating evidence indicates that targeted modulation of this pathway may alleviate neuroinflammation associated with SAE and enhance neurological recovery.

**Conclusion:**

Targeting the PI3K/Akt pathway represents a promising therapeutic approach for SAE. Advances in the development of specific agonists and inhibitors provide new opportunities for clinical translation and drug discovery in neuroinflammatory conditions.

## 1 Introduction

Sepsis is a systemic inflammatory response syndrome (SIRS) caused by infection, characterized by dysregulation of the body’s immune response to infection. It is one of the key causes of death in critically ill patients in the intensive care unit [1]. Among these, sepsis-associated encephalopathy (SAE) is one of the most common complications of sepsis, with an incidence rate as high as 70% [2], which significantly increases the morbidity and mortality rate and diminishes the quality of life of the patients [3,4]. The pathogenesis of SAE is complex and multifactorial, with contributing key factors including activation of microglia in the central nervous system (CNS), blood–brain barrier (BBB) dysfunction, brain edema, neurotransmitter dysfunction, impaired brain metabolism, and mitochondrial dysfunction [5–8]. Additional mechanisms, such as the accumulation of amyloid-β and tau proteins, activation of the complement system, and direct neuronal injury, may also contribute to the development of SAE [9]. Overall, SAE results from the synergistic effects of multiple factors, rather than a single cause. Among these, neuroinflammation has been shown to play a crucial role throughout the SAE process and is closely associated with prognosis.

Neuroinflammation is an immune response activated by microglia and astrocytes in the CNS, usually occurring in response to CNS injury, infection, toxin stimulation, or autoimmunity [9]. Systemic inflammation induced by sepsis is mediated by the excessive release of proinflammatory cytokines – such as interleukin-1β (IL-1β), tumor necrosis factor-α (TNF-α), and interleukin-6 (IL-6) – which increases the permeability of the BBB, thereby facilitating inflammatory factors to enter the brain and trigger an inflammatory response in the CNS. The sustained inflammatory response disrupts the BBB and facilitates the infiltration of peripheral immune cells. This exacerbates CNS injury, leading to neuronal damage [10,11], demyelination [12], impaired regeneration [13], and synaptic dysfunction.

Among the numerous molecular pathways implicated in neuroinflammation, the phosphatidylinositol 3-kinase/protein kinase B (PI3K/Akt) signaling pathway has been extensively studied for its involvement in various physiological processes within the CNS, including cell survival, autophagy, neurogenesis, neuronal proliferation and differentiation, synaptic plasticity, anti-apoptosis, anti-oxidative stress, and neural repair [9,14]. Recently, several studies have confirmed that this pathway is closely related to the development of neurological diseases and plays an important role in modulating various pathological changes [15–17].

Based on the roles of neuroinflammation and PI3K/Akt pathway activation in the pathogenesis of SAE, this article aims to elucidate their specific roles and interrelationships in SAE development. The following sections will comprehensively describe the neuroinflammation triggered by sepsis, the underlying mechanisms of the PI3K/Akt pathway, its role in SAE, and the potential treatment of SAE through targeting the PI3K/Akt pathway.

## 2 Sepsis-induced neuroinflammation

The presence of neuroinflammation in SAE has been clearly demonstrated [18,19]. Autopsy studies of patients who died from sepsis revealed significantly increased expression of markers associated with acute neuroinflammation, suggesting that neuroinflammation may play a critical role in the progression of SAE [20]. While infection does not occur directly in the brain, peripheral inflammatory signals can trigger widespread neuroinflammation through both neuronal and humoral pathways [21]. Neuroinflammation is initiated by multiple biological mechanisms, including immune responses, oxidative stress, the release of inflammatory mediators, and damage to the BBB. Although neuroinflammation can initially have a protective effect, prolonged or excessive inflammation can result in neural tissue damage.

Neuroinflammatory responses can be classified into two categories: secondary inflammatory responses induced by peripheral immune cells and primary inflammatory responses triggered by resident immune cells. Initially, during sepsis, pathogens or their associated toxins – such as lipopolysaccharide (LPS) – stimulate the host immune system, leading to an exaggerated inflammatory response and the subsequent development of SIRS. These peripheral inflammatory signals affect the CNS through two main pathways: humoral and neuronal. Through the humoral pathway, elevated circulating pro-inflammatory factors (TNF-α, IL-1β, and IL-6) enter the brain via the disrupted BBB and directly activate CNS inflammation. In a septic environment, the intense secretion of inflammatory factors and chemokines recruits peripheral immune cells, such as neutrophils and macrophages, to the brain, thereby exacerbating neuronal damage [22,23]. Neuroinflammation can also be amplified through neurotransmission, with the vagus nerve upregulating pro-inflammatory gene expression by transmitting signals to the nucleus tractus solitarius of the medulla oblongata [24]. This central-peripheral inflammation cascade amplifies the inflammatory response. In sepsis, microglia become hyperactivated, releasing large amounts of pro-inflammatory cytokines (TNF-α, IL-1β) and chemokines (MCP-1). Overactivated microglia induce apoptosis and synaptic damage in neurons, stimulate reactive oxygen species (ROS) production [25], and further trigger oxidative stress and inflammatory cascades. Astrocytes are also activated [26], and synergize with microglia to exacerbate the inflammatory response. This includes breaking down matrix components in the BBB, leading to barrier disruption, and secreting large quantities of inflammatory factors (IL-6), which promote the spread of inflammation. Cytokines and chemokines also disrupt the integrity of the BBB by affecting the expression of tight junction proteins (Claudin, Occludin) [22,23,27]. The sepsis-induced immune response produces an abundance of pro-inflammatory factors (TNF-α, IL-1β), which bind to specific receptors and activate apoptotic signaling pathways such as Fas and Caspase [28,29]. This activation ultimately leads to neuronal and glial cell death, causing further damage to the nervous system. Additionally, activated inflammatory mediators and immune cells generate ROS and reactive nitrogen species, leading to increased oxidative damage [30,31]. These free radicals damage cell membranes, proteins, and DNA, which further contribute to neuronal dysfunction, apoptosis, and long-term neurological impairment. Sepsis-induced oxidative stress also disrupts mitochondrial function, compromising the cellular energy supply. Mitochondrial damage exacerbates neuronal apoptosis and creates a vicious cycle by releasing cytokines and activating neuroinflammatory pathways [32].

## 3 PI3K/Akt pathway and its role in brain tissue

The PI3K/Akt signaling pathway consists of two main components: phosphatidylinositol 3 (PI3K) and its downstream serine/threonine protein kinase B (PKB, also known as Akt). PI3K is a class of intracellular lipid kinases, classified into types I, II, or III based on substrate specificity and sequence homology [33,34]. Akt is a proto-oncogene product that, upon activation, modulates a variety of downstream signaling molecules, including mammalian target of rapamycin (mTOR) [35] and glycogen synthase kinase-3 (GSK-3) [36], among others.

The PI3K/Akt signaling pathway is a central intracellular regulatory network involved in diverse biological processes, including cell survival, proliferation, metabolism, protein synthesis, immune regulation, and stress responses. By promoting proliferation, inhibiting apoptosis, and enhancing cell survival, this pathway plays a critical role in tumorigenesis and has emerged as a prominent target for cancer therapy [37–39]. Akt, a key effector of the PI3K/Akt pathway, regulates glucose and lipid metabolism, as well as cell differentiation and growth [40], and holds therapeutic potential for diabetes and metabolic disorders. The PI3K/Akt/mTOR pathway also plays a critical role in bone and joint diseases, such as osteoarthritis [41], and in erythroid hematopoiesis [40], suggesting its potential as a novel therapeutic target. In neurological disorders, the PI3K/Akt pathway regulates neuronal survival, mitigates inflammation, preserves BBB integrity, and exerts neuroprotective effects in conditions such as Alzheimer’s and Parkinson’s diseases [42,43]. Thus, the PI3K/Akt pathway holds broad clinical potential across various diseases, including cancer, metabolic disorders, neurological conditions, and osteoarticular diseases.

In normal brain tissues, the PI3K/Akt signaling pathway plays a crucial role in neuronal survival, synaptic plasticity, energy metabolism, BBB integrity, and neurodevelopment [44]. The activation of this pathway depends on upstream ligands such as derived brain-derived neurotrophic factor (BDNF) and insulin-like growth factor 1 (IGF-1), and mediates multiple regulatory effects on neuronal anti-apoptosis, resistance to oxidative stress, synaptic plasticity, and energy metabolism through downstream targets including mTOR, GSK-3β, and cAMP response element-binding protein 2 [45]. For example, Akt inhibits the mitochondrial apoptotic pathway by phosphorylating pro-apoptotic proteins such as BAD and Caspase-9, thereby preventing premature neuronal apoptosis and contributing to the maintenance of the functional integrity of brain tissue [46]. Akt exerts antioxidant effects by activating the Nrf2 pathway and scavenging ROS [47]. It also activates mTOR-dependent protein synthesis to support dendritic spine formation and long-term potentiation (LTP) [47], and promotes the membrane translocation of the glucose transporter GLUT4 to enhance neuronal glucose uptake. In addition, Akt plays a key role in stabilizing the intracerebral microenvironment by maintaining the expression of endothelial tight junction proteins, such as Zonula Cccludens-1 (ZO-1), at the BBB [48].

However, in the pathological state of SAE, the systemic inflammatory response and oxidative stress lead to inhibition of the PI3K/Akt signaling pathway. Proinflammatory cytokines, such as TNF-α and IL-1β, released during sepsis indirectly suppress the activity of the PI3K catalytic subunit p110 via activation of the nuclear factor kappa B (NF-κB) pathway [25]. Simultaneously, excessive ROS promote the conversion of phosphatidylinositol (3,4,5)-trisphosphate (PIP3) to phosphatidylinositol 4,5-bisphosphate (PIP2) by activating the lipid phosphatase PTEN, a negative regulator of PI3K, thereby impairing Akt membrane localization and phosphorylation [49]. Mitochondrial dysfunction further exacerbates energy metabolism disorders and reduces the efficiency of Akt activation [25]. Akt inactivation results in the dephosphorylation of BAD, which in turn activates Bax/Bak-mediated mitochondrial cytochrome c release and triggers the caspase cascade, ultimately enhancing neuronal apoptosis. Additionally, GSK-3β disinhibition leads to Tau protein hyperphosphorylation, promoting the formation of neurofibrillary tangles and downregulating the expression of synapse-associated proteins such as PSD-95, contributing to synaptic damage [50]. Decreased Akt activity in endothelial cells also compromises tight junction integrity, aggravating brain edema and neuroinflammation.

**Figure 1:**
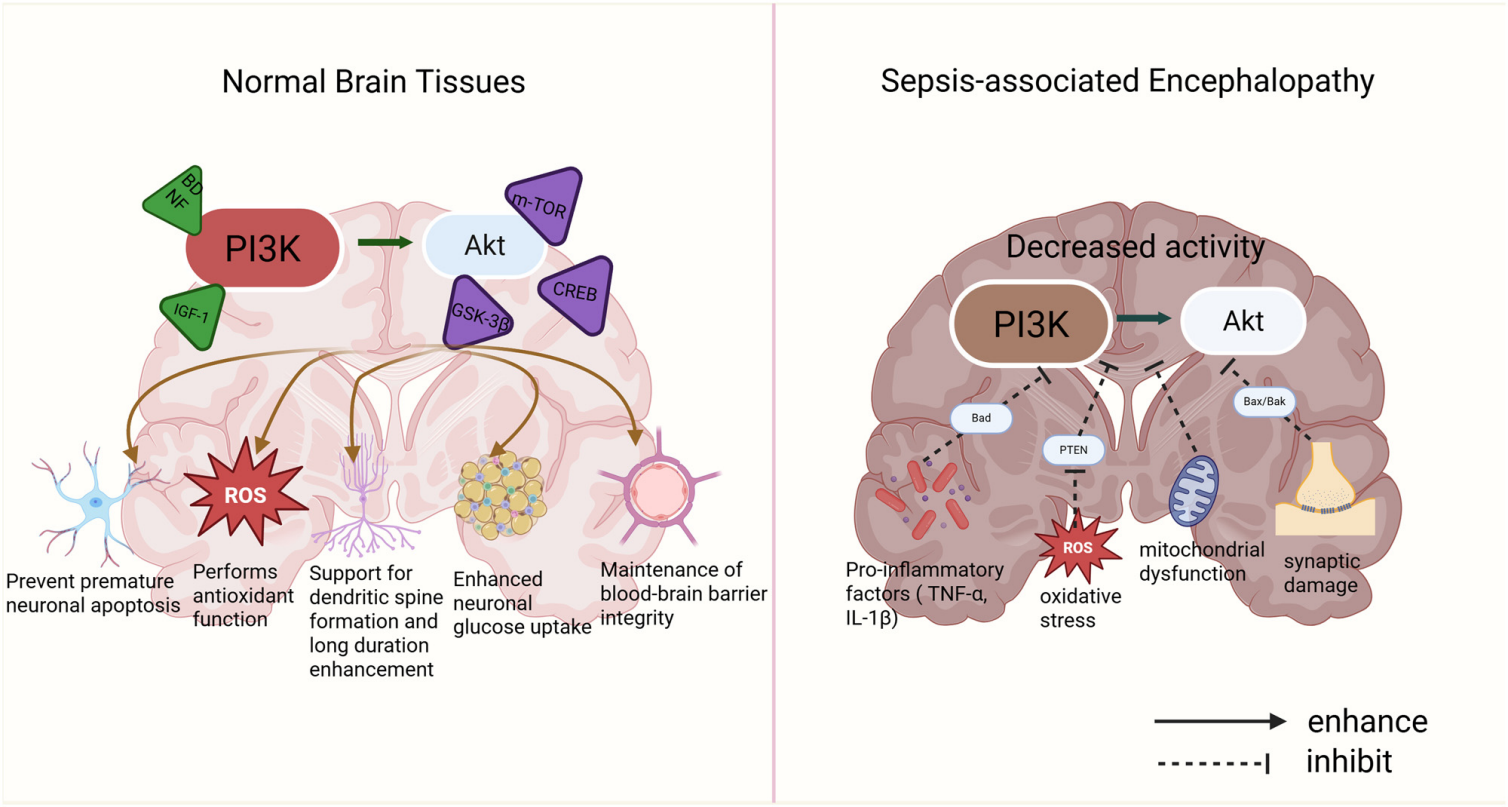
Differences in PI3K/Akt pathway in normal brain tissue and SAE conditions. ROS: reactive oxygen species.

In summary, the PI3K/Akt pathway plays a critical role in maintaining CNS homeostasis under normal conditions. However, in SAE, its expression and activity are markedly altered, resulting in the loss of its original protective functions and, in some cases, contributing to disease progression. Comparing the dynamic changes of this pathway between physiological and pathological states may help elucidate its role in SAE pathogenesis and provide a theoretical foundation for targeted therapeutic interventions. (Figure 1).

## 4 PI3K/Akt pathway and neuroinflammation in SAE

As discussed previously, sepsis-induced neuroinflammation is a core pathogenic mechanism of SAE. Within the pathophysiological framework of SAE, the PI3K/Akt pathway plays a dual regulatory role in both CNS injury and neuroprotection. It does so by modulating the expression of neuroinflammatory factors, regulating microglial activation, maintaining the integrity of the BBB, and controlling neuronal apoptosis. The PI3K/Akt pathway is a classical signaling cascade that regulates neuronal survival and neurogenesis, making it a crucial player in the progression and potential treatment of SAE [40,51]. In the CLP mouse model, protein expression levels of PI3K and Akt in hippocampal tissues were significantly decreased [52–54], whereas activation of the PI3K/Akt pathway attenuated SAE-related damage [52–57].

### 4.1 Regulation of inflammatory factors

The PI3K/Akt pathway suppresses the excessive release of pro-inflammatory cytokines during sepsis through multiple mechanisms. First, it initiates a negative feedback regulatory loop that significantly reduces the overexpression and secretion of pro-inflammatory cytokines, including TNF-α, IL-1β, and IL-6 [58–60]. Second, the pathway upregulates the expression of anti-inflammatory mediators, such as IL-10, which subsequently inhibits pro-inflammatory cytokine production via paracrine signaling [58]. Additionally, the PI3K/Akt pathway inhibits the activation and nuclear translocation of NF-κB, further modulating inflammation [61]. It also contributes to the regulation of the Th1/Th2 cytokine balance [62], thereby attenuating the inflammatory cascade. Clinical studies have confirmed that pharmacological agents like dexmedetomidine [63] and metformin [64] can activate the PI3K/Akt pathway, inhibiting pro-inflammatory cytokine release and improving sepsis-associated brain and lung injury.

### 4.2 Regulation of microglia polarization

The PI3K/Akt signaling pathway plays a crucial role in regulating microglia polarization. It modulates microglial M1/M2 polarization by influencing multiple signaling pathways [65], including NF-κB [66] and chemokine receptor CXCR7 [67], which promote the transformation of microglia to the M2 phenotype and enhance their anti-inflammatory properties. M2-polarized microglia secrete anti-inflammatory cytokines, such as IL-10 and TGF-β [68], thereby reducing the neuroinflammatory response. Agents like tretinoin and curcumin have been demonstrated to inhibit the activation of pro-inflammatory M1 microglia while promoting the anti-inflammatory properties of M2 microglia, presumably via activation of the PI3K/Akt pathway [69]. Furthermore, the PI3K/Akt pathway indirectly regulates microglial inflammation by modulating the neuron-derived BDNF-PI3K/Akt signaling axis [70,71]. Akt increases the secretion of BDNF from neurons by enhancing vesicular transport, which in turn strengthens the inhibitory effect of neurons on microglial inflammation. Compounds like pineoside [72] and paeoniflorin [72] regulate the BDNF-PI3K/Akt axis, achieving a balanced regulation of microglia M1/M2 polarization and alleviating neuroinflammatory disorders such as SAE.

### 4.3 Maintenance of BBB integrity

The PI3K/Akt pathway plays a crucial role in maintaining the integrity of the BBB. Akt regulates the expression of tight junction proteins, such as Claudin, Occludin, and ZO-1, through the activation of the PI3K/Akt pathway, thereby preserving BBB structural integrity [73,74]. Chen et al. [75] demonstrated in an *in vitro* BBB model that activation of the PI3K/Akt pathway enhances the transcription and translation of tight junction proteins, resulting in decreased permeability of endothelial cell monolayers. Wang et al. [76] found that in a murine model of sepsis treatment with a PI3K-selective agonist significantly increased the expression of tight junction proteins in brain microvascular endothelial cells. Additionally, the PI3K/Akt pathway inhibits the TNF-α- and IL-1β-induced expression of matrix metalloproteinases, thereby reducing extracellular matrix degradation and protecting the BBB structure [77]. Zhi et al. [78] observed that selective PI3K agonist treatment significantly reduced levels of S100β and neuron-specific enolase in the cerebrospinal fluid of sepsis patients, along with other markers of BBB damage, and improved neurocognitive function scores. Gong et al. [79] reported that combined administration of a PI3K agonist and an anti-inflammatory agent significantly reduced the morbidity and mortality of patients with SAE, and the levels of tight junction protein degradation products, such as Occludin and Claudin-5 fragments, were notably lower in the peripheral blood of treated patients. This suggests that the combination regimen may exert neuroprotective effects by preserving BBB integrity.

### 4.4 Regulation of neuronal apoptosis

Neuronal apoptosis is a key pathological feature in sepsis-induced neuroinflammation, with its regulatory mechanisms linked to the PI3K/Akt pathway. First, the PI3K/Akt pathway inhibits neuronal apoptosis by regulating several downstream effector molecules. Activated Akt effectively blocks Bad-induced apoptosis by phosphorylating Bad at the Ser136 site, preventing its binding to Bcl-2. Additionally, the pathway upregulates the nuclear translocation of the antioxidant-related transcription factor Nrf2, which increases the expression of superoxide dismutase and catalase, thereby reducing oxidative stress-induced neuronal injury. Puerarin has been shown to reduce neuronal apoptosis by inhibiting oxidative stress through the PI3K/Akt/Nrf2 pathway [80]. Furthermore, the PI3K/Akt pathway promotes neural repair and regeneration. Akt-mTOR signaling enhances myelin sheath growth and stability during development by driving cap-dependent translation to facilitate myelin formation [81]. It has also been demonstrated that resveratrol can activate PI3K/Akt signaling to promote axonal regeneration and neurological recovery. Additionally, the PI3K/Akt pathway may play a role in regulating synaptic plasticity by modulating the reorganization of post-synaptic proteins, such as PSD-95 [82], and influences synaptic transmission efficiency as well as LTP [83]. Moreover, novel PI3K agonists have shown promising neuroprotective effects in animal models, providing new intervention targets for the treatment of SAE [84].

### 4.5 Discovery of new molecules

**Figure 2:**
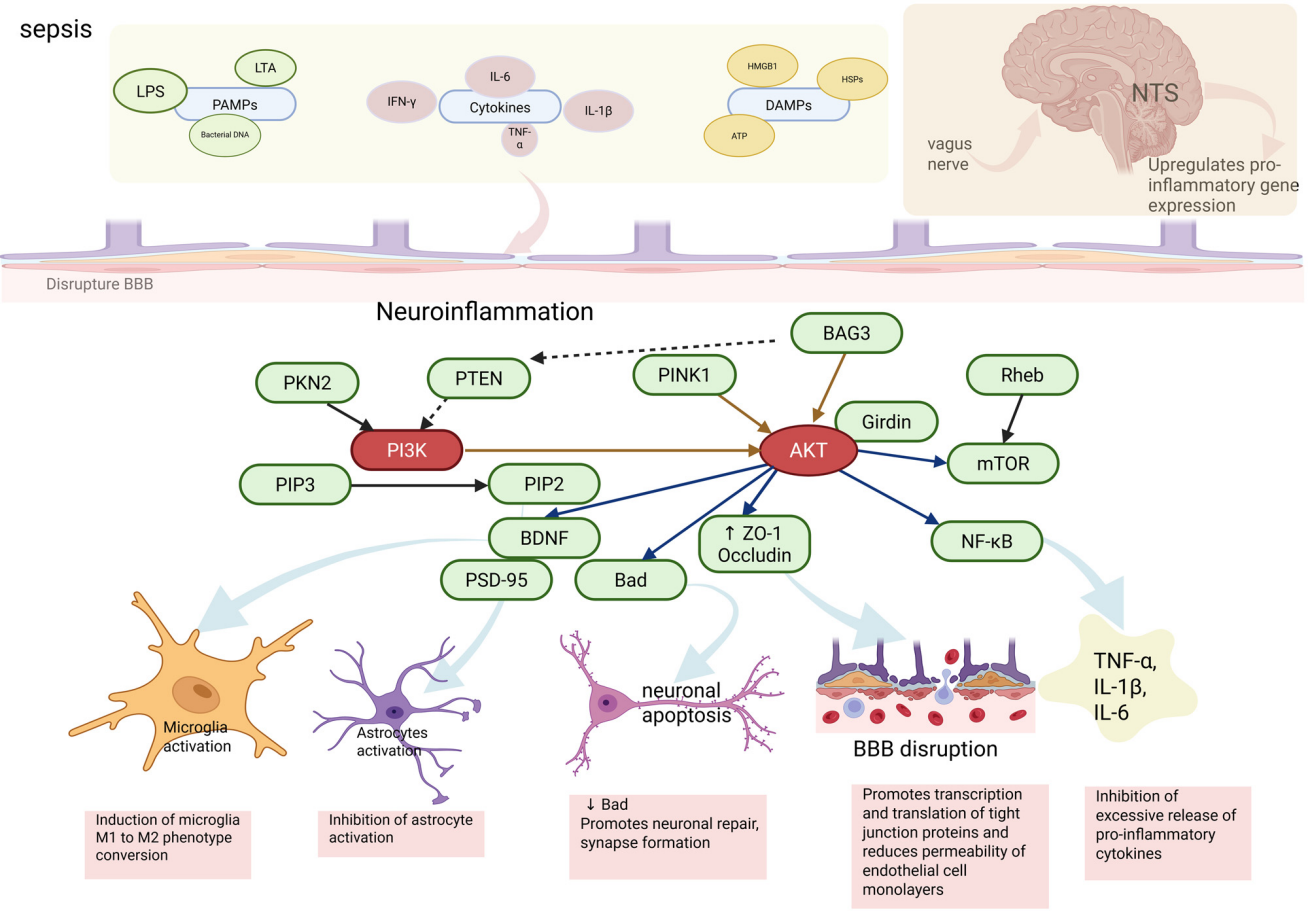
Inflammatory signaling regulates SAE through the PI3K/Akt pathway. PAMPs: pathogen-associated molecular patterns; DAMPS: damage-associated molecular patterns; LTA: lipoteichoic acid; NTS: nucleus tractus solitarius; BBB: blood–brain barrier.

In recent years, several novel molecules have been identified as regulators of the PI3K/Akt pathway, with significant effects on the development and progression of SAE. Protein kinase N2 (PKN2), in particular, exerts a broad range of regulatory effects on the PI3K/Akt signaling pathway through mechanisms such as direct phosphorylation, modulation of membrane localization, and catalytic activity. PKN2 can directly phosphorylate the p85 regulatory subunit of PI3K [85], inducing a conformational change that alters its interactions with other signaling molecules. Furthermore, PKN2 affects the membrane localization of PI3K, thereby modulating its enzymatic activity [85]. Wang et al. [86] demonstrated that PKN2 overexpression activated the mTOR pathway in PC12 cells, reducing H_2_O_2_-induced oxidative damage and apoptosis. Additionally, PKN2 modulates Akt activity by phosphorylating its Ser473 site [87], a key step in regulating the function of Akt within the PI3K/Akt pathway. By phosphorylating Akt at this critical site, PKN2 influences the activity and substrate specificity of Akt, thereby affecting downstream signaling processes. In addition, PKN2 may play a role in regulating the integrity of the BBB [88]. Bai et al. [89] found that H_2_ alleviates septic brain injury by activating PKN2 phosphorylation, which is associated with the PI3K pathway. While PKN2 exerts a broad range of regulatory effects on the PI3K/Akt signaling pathway, there are currently fewer studies focusing on its role in septic encephalopathy. This presents an opportunity for future, more in-depth research. PTEN, as a negative regulator of the PI3K/Akt pathway, inhibits AKT activation by dephosphorylating PIP3 and converting it to PIP2 [90]. This action helps maintain normal neuronal function and survival, protecting against neuronal injury caused by dysregulated signaling. Furthermore, the signaling between PTEN-induced kinase 1 (PINK1) and the Parkin pathway is crucial in SAE. High expression of PINK1 can activate mitochondrial autophagy through up-regulation of Parkin, which reduces inflammatory responses, preventing neuroinflammation and alleviating cognitive impairments in SAE mice. In the absence of PINK1, there is a suppression of Ca^2+^ transients in the hippocampus, leading to elevated intracellular Ca^2+^ levels, which exacerbate sepsis-induced cognitive dysfunction in mice, highlighting the vital role of PINK1 in maintaining neuronal stability [91]. Girdin can inhibit the production of pro-inflammatory cytokines, promote neuronal survival, and prevent apoptosis by regulating the activation of the PI3K/Akt pathway, thereby alleviating neuroinflammation [92,93]. Phosphorylated Girdin enhances the activity of intracellular anti-apoptotic factors, such as Bcl-2 and mTOR, which reduce programmed cell death [94]. Additionally, Girdin regulates microglial activation, as overactivation of microglia exacerbates the neuroinflammatory response. Rheb (Ras homolog enriched in brain), a small GTP-binding protein, plays a key role in the PI3K/Akt/mTOR signaling pathway by directly activating mTOR, which is crucial for neuroprotection. In an LPS-induced neuroinflammation model, upregulation of Rheb was linked to astrocyte proliferation and neuronal apoptosis [95]. The Rheb-mTOR signaling pathway contributes to neuroinflammation-induced astrocyte activation and neuronal apoptosis through cell cycle activation [96]. Moreover, specific overexpression of Rheb in retinal ganglion cells significantly reduced cell death and effectively induced axonal regeneration [97], suggesting that Rheb promotes neural repair, potentially alleviating sepsis-induced neuroinflammation. Additionally, BAG3 is involved in regulating Akt downstream targets like mTORC1, affecting cellular autophagy processes. BAG3 forms a complex with CHIP (C-terminus of Hsc70 interacting protein) [98], which facilitates the ubiquitination and degradation of key inhibitors negatively regulating PI3K/Akt signaling, such as phosphatase and Tensin homolog deleted on chromosome 10 (PTEN), thereby positively regulating this pathway. Bcl-2-associated athanogene 3 (BAG3) also plays a protective role in sepsis-induced acute kidney injury (AKI), and studies have shown that sevelamer sodium can attenuate AKI in a rat sepsis model through inhibition of the PI3K/Akt pathway, with BAG3 involved in this protective mechanism. Although there are currently fewer studies directly examining these molecules in SAE, they play critical roles in the PI3K/Akt signaling pathway, which is integral to SAE pathogenesis. Future research should focus on how these molecules specifically contribute to SAE and whether they can be targeted for effective therapeutic interventions, potentially offering more effective treatments for patients with SAE (Figure 2).

## 5 Therapeutic potential of PI3K/Akt pathway modulators

IGF-1 attenuates the inflammatory response and ameliorates SAE by activating the PI3K/Akt pathway. Treatment with recombinant human IGF-1 significantly reduced serum levels of IL-1β, TNF-α, and IL-6 in patients with SAE, while also improving the Glasgow Coma Score (GCS), strengthening BBB integrity, reducing neuroinflammation, and enhancing cognitive function [99,100]. BDNF activates the PI3K/Akt signaling pathway through the TrkB receptor, upregulates the expression of the anti-apoptotic protein Bcl-2, and promotes the expression of synaptic plasticity-associated proteins (PSD95) [101,102]. BDNF has been shown to significantly ameliorate memory deficits in a mouse model of sepsis and reduce the expression of pro-inflammatory cytokines in the CNS. Several studies have shown that various naturally active compounds exert neuroprotective effects by modulating the PI3K/Akt signaling pathway. Curcumin significantly increased the level of phosphorylated Akt in neurons (the p-Akt/Akt ratio increased 2.1-fold), promoting the nuclear translocation of Nrf2 and enhancing cellular resistance to oxidative stress [103]. Resveratrol significantly reduced the volume of cerebral infarcts and neurological damage scores in cerebral ischemia/reperfusion rats, significantly lowering levels of myeloperoxidase, TNF-α, and upregulating p-Akt expression. The use of Akt inhibitors blocked the effects of resveratrol. Resveratrol was also shown to reduce neuronal apoptosis, as evidenced by an increase in the Bcl-2/Bax ratio and a decrease in the number of TUNEL-positive apoptotic cells [104]. Additionally, quercetin and rhodiola rosea glycosides synergistically activate PI3K/Akt downstream targets, significantly reducing pro-inflammatory factors and improving cognitive function [105,106]. Oleanolic acid [107] and dihydromyricetin (DHM) [108] inhibit oxidative stress through activation of the PI3K/Akt pathway, thereby exerting neuroprotective effects. Based on their role in other neuroinflammatory diseases, these natural compounds may have important therapeutic potential in SAE. In cases of overactivated PI3K/Akt signaling, the PI3K-selective inhibitor LY294002 exerts a bidirectional modulatory effect, reducing inflammatory responses and neurotoxicity [104,109]. However, the targets of PI3K inhibitors need to be carefully selected to avoid inhibiting neuroprotective effects.

Several PI3K inhibitors have been developed for tumor therapy, such as PI-103, BKM120, and GDC-0941 [110,111]. These inhibitors block tumor cell proliferation and survival by targeting key nodes of the PI3K/Akt pathway, including PI3K, Akt, and mTOR. Some PI3K inhibitors have been approved for the treatment of specific hematologic malignancies. For example, Linperlisib, the first highly selective PI3Kδ inhibitor marketed in China, has been approved for the treatment of relapsed and/or refractory follicular lymphoma (FL), demonstrating significant clinical efficacy and good tolerability [112]. In addition, Duvelisib [113], a dual PI3Kδ and PI3Kγ inhibitor, has also been approved by the FDA for the treatment of adult patients with relapsed/refractory chronic lymphocytic leukemia (CLL), small lymphocytic lymphoma (SLL), and FL who have received at least two prior therapies. Although these drugs have shown effectiveness in treating specific types of hematologic malignancies, their potential application in neuroinflammatory and neurological disorders requires further research and exploration. Although most compounds targeting the PI3K/Akt pathway – such as estrogen [114], resveratrol, curcumin [115], GDC-0941, duvelisib, and leniolisib [112] – have already entered clinical trials for other diseases (oncology, metabolic disorders, and neurodegenerative diseases), their clinical application in sepsis, especially SAE, remains largely unexplored or is still in its infancy. This suggests that, in the future, compounds with established clinical safety profiles could be prioritized for translational research in SAE, particularly through the use of animal models to verify their target specificity and neuroprotective efficacy (Table 1).

**Table 1:** Major research models, mechanisms, and key findings of PI3K/Akt pathway agonists and inhibitors associated with SAEs

Compound	Mechanisms	Model	Key findings	Ref.
Recombinant human IGF-1	Activates PI3K/Akt pathway to reduce inflammation	C57BL/6 mice; sepsis induced by intratracheal instillation of *P. aeruginosa* (PA103 strain)	Decrease in serum IL-1β, TNF-α, IL-6 levels; increase in GCS score; enhancement of BBB function; decrease in neuroinflammation and improvement in cognitive ability	[99,100]
BDNF	Activates the PI3K/Akt pathway via TrkB receptors	SCID-Beige mice; NB xenograft model for metastasis	Improvement of memory deficits and reduction of pro-inflammatory cytokines in CNS	[101,102]
Estrogen	Activates PI3k/Akt, upregulates Bcl-2, and downregulates Bax and Caspase-3,8	VSC4.1 motor neuron cells	Antiapoptotic	[116]
Curcumin	Increases phosphorylated Akt (p-Akt) and promotes Nrf2 nuclear translocation	C57BL/6 mice; LPS-induced sepsis model; HL-1 cardiomyocytes; LPS stimulation	Enhanced antioxidant stress response and 2.1-fold increase in p-Akt/Akt ratio, promoting neuroprotection	[103]
Resveratrol	Increases p-Akt expression and inhibits oxidative stress	SD rats; MCAO-induced cerebral I/R injury model	Reduced cerebral infarct volume, improved neurologic scores, decreased MPO, TNF-α, increased p-Akt, and decreased apoptosis	[117]
Quercetin	Activates PI3K/Akt pathway, anti-inflammatory effects	C57BL/6 mice; *G. parasuis*-induced encephalitis model; mouse brain endothelial cells; *G. parasuis* stimulation	Significantly reduced inflammatory cytokines and improved cognitive function	[105,106]
Oleanolic acid	Activates PI3K/Akt pathway to inhibit oxidative stress and neuroinflammation	Rats; methylmercury-induced ALS model	Reduces oxidative stress and neuroinflammation	[107]
DHM	Activates the PI3K/Akt pathway to reduce oxidative stress	C57BL/6 mice; MPTP-induced Parkinson’s disease model; MES23.5 cells; MPP⁺ stimulation	Reduces oxidative stress and neuroinflammation and provides neuroprotection	[108]
PI-103 series	PI3K inhibitor targeting the PI3K/Akt/mTOR axis	Human tumor xenograft models (glioblastoma, breast cancer, leukemia, prostate cancer, etc.)	Inhibits tumor cell proliferation and survival by targeting the PI3K/Akt/mTOR pathway	[110,111]
BKM120 series	PI3K inhibitor targeting the PI3K/Akt pathway	Human tumor xenograft models (glioblastoma, breast cancer, leukemia, prostate cancer, etc.)	Inhibits tumor cell growth and survival by blocking PI3K/Akt signaling	[110,111]
GDC-0941	PI3K inhibitor targeting the PI3K/Akt pathway	Human tumor xenograft models (glioblastoma, breast cancer, leukemia, prostate cancer, etc.)	Inhibits tumor growth, metastasis, and survival in preclinical models by blocking PI3K/Akt signaling	[110,111]
Imperius	Highly selective PI3Kδ inhibitor	Refractory/relapsed FL	Approved for FL treatment in China; demonstrated significant clinical efficacy and tolerability	[112]
Duvelisib	Dual PI3Kδ/PI3Kγ inhibitor	Refractory CLL and FL	FDA approved for the treatment of CLL, SLL, and FL; effective in patients with relapsed/refractory disease	[113]
LY294002	PI3K inhibitor targeting the PI3K pathway	Rat astrocyte; LPS-induced astrocyte culture model used	Inhibits the PI3K/Akt pathway; shows bidirectional regulation in overactive pathways, reducing inflammation and neurotoxicity	[104,109]

## 6 Conclusion

Neuroinflammation is a key contributor to the pathogenesis of SAE, leading to CNS injury and adversely affecting cognitive function and patient survival. The underlying mechanisms primarily involve the activation of microglia and astrocytes, the release of pro-inflammatory cytokines and chemokines, disruption of the BBB structure, increased oxidative stress, and mitochondrial dysfunction. Among the numerous signaling pathways implicated in SAE, the PI3K/Akt pathway has emerged as a central regulator. This pathway plays a pivotal role in modulating neuroinflammatory responses, preserving BBB integrity, and promoting neuronal survival. Several studies have demonstrated that activation of the PI3K/Akt pathway by specific compounds can attenuate inflammation and neuronal damage, thereby alleviating SAE-related symptoms. However, the majority of these findings remain at the preclinical stage, and no PI3K/Akt-targeted agents have yet demonstrated clinical efficacy in SAE treatment.

As mentioned above, the PI3K/Akt signaling pathway plays a key role in regulating neuroinflammation, apoptosis, and BBB function, and holds great promise for clinical application. Activation of this pathway can reduce brain tissue damage and improve cognitive function, making it applicable to a variety of neurological diseases, including SAE. In addition, the PI3K/Akt pathway is closely related to immune system homeostasis. Moderate activation of this pathway can inhibit excessive immune responses and reduce systemic inflammation, which is particularly important in SAE, a condition characterized by a pathological “inflammatory storm.” Currently, several drugs targeting this pathway have entered clinical trials for oncology and metabolic diseases and have demonstrated favorable safety profiles. As a potential therapeutic target, the PI3K/Akt pathway is expected to offer a novel intervention strategy for diseases such as SAE in the future.

Despite these advances, several critical knowledge gaps remain. The PI3K family comprises multiple isoforms – PI3Kα, PI3Kγ, and PI3Kδ – each with distinct cellular distribution and functions. For example, PI3Kγ, as a central driver of microglial inflammatory responses, promotes M1-type polarization through the activation of chemokine receptors (the CXCL12–CXCR4 axis), thereby inducing NLRP3 inflammasome activation and the infiltration of peripheral immune cells into the brain [118]. PI3Kα, on the one hand, maintains neuronal metabolism and anti-apoptotic functions via the Akt/mTOR pathway [119]; on the other hand, its overactivation may exacerbate mitochondrial autophagy disorders due to oxidative stress [120]. Current research on the roles of these isoforms in SAE is limited, fragmented, and lacks systematic comparison and precise localization. Moreover, most studies have been conducted in acute animal models or *in vitro* systems, and their clinical relevance and translational potential remain to be validated. Notably, the PI3K/Akt pathway is intricately linked with multiple other signaling cascades – including MAPK, JAK/STAT, NF-κB, mTOR, and NLRP3 – which can induce diverse, and sometimes opposing, biological outcomes. This complexity makes it challenging to predict the net effect of pathway activation or inhibition in the inflammatory milieu of SAE.

Future studies should aim to delineate the cell-type-specific and stage-specific roles of individual PI3K isoforms during SAE progression and evaluate their potential as therapeutic targets in terms of efficacy and safety. Additionally, integrating single-cell transcriptomics and multi-omics approaches may provide insights into the dynamic crosstalk between the PI3K/Akt pathway and other signaling networks. On this basis, the development of selective, brain-targeted PI3K modulators, and the investigation of combination therapies – such as anti-inflammatory agents paired with BBB-protective compounds – may accelerate the translation of preclinical findings into clinically viable interventions for SAE.

## References

[1] Rhodes A, Evans LE, Alhazzani W, Levy MM, Antonelli M, Ferrer R, et al. Surviving sepsis campaign: international guidelines for management of sepsis and septic shock: 2016. Intensive Care Med. 2017;43(3):304–77. 10.1007/s00134-017-4683-6. 28101605

[2] Czempik PF, Pluta MP, Krzych ŁJ. Sepsis-associated brain dysfunction: a review of current literature. Int J Environ Res Public Health. 2020;17(16):5852. 10.3390/ijerph17165852. PMC7460246 32806705

[3] Pan S, Lv Z, Wang R, Shu H, Yuan S, Yu Y, et al. Sepsis-induced brain dysfunction: pathogenesis, diagnosis, and treatment. Oxid Med Cell Longev. 2022;2022:1328729. 10.1155/2022/1328729. PMC9433216 36062193

[4] Pu Y, Zhao L, Xi Y, Xia Y, Qian Y. The protective effects of mirtazapine against lipopolysaccharide (lps)-induced brain vascular hyperpermeability. Bioengineered. 2022;13(2):3680–93. 10.1080/21655979.2021.2024962. PMC8973832 35081868

[5] Zhou Y, Bai L, Tang W, Yang W, Sun L. Research progress in the pathogenesis of sepsis-associated encephalopathy. Heliyon. 2024;10(12):e33458. 10.1016/j.heliyon.2024.e33458 PMC11254713 39027435

[6] Hong Y, Chen P, Gao J, Lin Y, Chen L, Shang X. Sepsis-associated encephalopathy: from pathophysiology to clinical management. Int Immunopharmacol. 2023;124(Pt A):110800. 10.1016/j.intimp.2023.110800. 37619410

[7] Wang X, Wen X, Yuan S, Zhang J. Gut–brain axis in the pathogenesis of sepsis-associated encephalopathy. Neurobiol Dis. 2024;195:106499. 10.1016/j.nbd.2024.106499. 38588753

[8] Xin Y, Tian M, Deng S, Li J, Yang M, Gao J, et al. The key drivers of brain injury by systemic inflammatory responses after sepsis: microglia and neuroinflammation. Mol Neurobiol. 2023;60(3):1369–90. 10.1007/s12035-022-03148-z PMC9899199 36445634

[9] Long LH, Cao WY, Xu Y, Xiang YY. Research progress on the role of microglia in sepsis-associated encephalopathy. Sheng Li Xue Bao. 2024;76(2):289–300. 38658377

[10] Chen Y, Qin C, Huang J, Tang X, Liu C, Huang K, et al. The role of astrocytes in oxidative stress of central nervous system: a mixed blessing. Cell Proliferation. 2020;53(3):e12781. 10.1111/cpr.12781 PMC7106951 32035016

[11] Yang QQ, Zhou JW. Neuroinflammation in the central nervous system: symphony of glial cells. Glia. 2019;67(6):1017–35. 10.1002/glia.23571. 30548343

[12] Leitner GR, Wenzel TJ, Marshall N, Gates EJ, Klegeris A. Targeting toll-like receptor 4 to modulate neuroinflammation in central nervous system disorders. Expert Opin Ther Targets. 2019;23(10):865–82. 10.1080/14728222.2019.1676416. 31580163

[13] Yong HYF, Rawji KS, Ghorbani S, Xue M, Yong VW. The benefits of neuroinflammation for the repair of the injured central nervous system. Cell Mol Immunol. 2019;16(6):540–6. 10.1038/s41423-019-0223-3 PMC6804643 30874626

[14] Matsuda S, Ikeda Y, Murakami M, Nakagawa Y, Tsuji A, Kitagishi Y. Roles of PI3K/AKT/GSK3 pathway involved in psychiatric illnesses. Diseases. 2019;7(1):22. 10.3390/diseases7010022 PMC6473240 30781836

[15] Ji Y, Wang D, Zhang B, Lu H. Bergenin ameliorates MPTP-induced Parkinson’s disease by activating PI3K/AKT signaling pathway. J Alzheimers Dis. 2019;72(3):823–33. 10.3233/jad-190870. 31658061

[16] Abdel Rasheed NO, Ibrahim WW. Telmisartan neuroprotective effects in 3-nitropropionic acid huntington’s disease model in rats: cross talk between PPAR-γ and PI3K/AKT/GSK-3β pathway. Life Sci. 2022;297:120480. 10.1016/j.lfs.2022.120480. 35278421

[17] Yoon JH, Lee N, Youn K, Jo MR, Kim HR, Lee DS, et al. Dieckol ameliorates aβ production via PI3K/AKT/GSK-3β regulated app processing in sweapp n2a cell. Mar Drugs. 2021;19(3):152. 10.3390/md19030152 PMC8001366 33804171

[18] Yin XY, Tang XH, Wang SX, Zhao YC, Jia M, Yang JJ, et al. Hmgb1 mediates synaptic loss and cognitive impairment in an animal model of sepsis-associated encephalopathy. J Neuroinflammation. 2023;20(1):69. 10.1186/s12974-023-02756-3 PMC10007818 36906561

[19] Zhang Y, Chen S, Tian W, Zhu H, Li W, Dai W, et al. Emerging trends and hot spots in sepsis-associated encephalopathy research from 2001 to 2021: a bibliometric analysis. Front Med (Lausanne). 2022;9:817351. 10.3389/fmed.2022.817351 PMC8918530 35295600

[20] Singer BH, Dickson RP, Denstaedt SJ, Newstead MW, Kim K, Falkowski NR, et al. Bacterial dissemination to the brain in sepsis. Am J Respir Crit Care Med. 2018;197(6):747–56. 10.1164/rccm.201708-1559OC PMC5855074 29232157

[21] Ren C, Yao RQ, Zhang H, Feng YW, Yao YM. Sepsis-associated encephalopathy: a vicious cycle of immunosuppression. J Neuroinflammation. 2020;17(1):14. 10.1186/s12974-020-1701-3 PMC6953314 31924221

[22] Huang X, Hussain B, Chang J. Peripheral inflammation and blood–brain barrier disruption: effects and mechanisms. CNS Neurosci Ther. 2021;27(1):36–47. 10.1111/cns.13569 PMC7804893 33381913

[23] Galea I. The blood–brain barrier in systemic infection and inflammation. Cell Mol Immunol. 2021;18(11):2489–501. 10.1038/s41423-021-00757-x PMC8481764 34594000

[24] Namgung U, Kim KJ, Jo BG, Park JM. Vagus nerve stimulation modulates hippocampal inflammation caused by continuous stress in rats. J Neuroinflammation. 2022;19(1):33. 10.1186/s12974-022-02396-z PMC8812005 35109857

[25] Moraes CA, Zaverucha-do-Valle C, Fleurance R, Sharshar T, Bozza FA, d’Avila JC. Neuroinflammation in sepsis: molecular pathways of microglia activation. Pharmaceuticals. 2021;14(5):416. 10.3390/ph14050416 PMC8147235 34062710

[26] Danielski LG, Giustina AD, Gava FF, Barichello T, Petronilho F. The many faces of astrocytes in the septic brain. Mol Neurobiol. 2022;59(12):7229–35. 10.1007/s12035-022-03027-7. 36136265

[27] Rempe RG, Hartz AMS, Bauer B. Matrix metalloproteinases in the brain and blood–brain barrier: versatile breakers and makers. J Cereb Blood Flow Metab. 2016;36(9):1481–507. 10.1177/0271678X16655551 PMC5012524 27323783

[28] Greene C, Connolly R, Brennan D, Laffan A, O'Keeffe E, Zaporojan L, et al. Blood-brain barrier disruption and sustained systemic inflammation in individuals with long covid-associated cognitive impairment. Nat Neurosci. 2024;27(3):421–32. 10.1038/s41593-024-01576-9 PMC10917679 38388736

[29] Han Y, Qiu L, Wu H, Song Z, Ke P, Wu X. Focus on the cgas-sting signaling pathway in sepsis and its inflammatory regulatory effects. J Inflamm Res. 2024;17:3629–39. 10.2147/JIR.S465978 PMC11162626 38855170

[30] Olufunmilayo EO, Gerke-Duncan MB, Holsinger RMD. Oxidative stress and antioxidants in neurodegenerative disorders. Antioxidants. 2023;12(2):517. 10.3390/antiox12020517 PMC9952099 36830075

[31] Geloso MC, Zupo L, Corvino V. Crosstalk between peripheral inflammation and brain: focus on the responses of microglia and astrocytes to peripheral challenge. Neurochem Int. 2024;180:105872. 10.1016/j.neuint.2024.105872. 39362496

[32] Zhang J, Chen S, Hu X, Huang L, Loh P, Yuan X, et al. The role of the peripheral system dysfunction in the pathogenesis of sepsis-associated encephalopathy. Front Microbiol. 2024;15:1337994. 10.3389/fmicb.2024.1337994 PMC10828041 38298892

[33] Sharma A, Mehan S. Targeting PI3K-AKT/mTOR signaling in the prevention of autism. Neurochem Int. 2021;147:105067. 10.1016/j.neuint.2021.105067. 33992742

[34] Cianciulli A, Porro C, Calvello R, Trotta T, Lofrumento DD, Panaro MA. Microglia mediated neuroinflammation: focus on PI3K modulation. Biomolecules. 2020;10(1):137. 10.3390/biom10010137 PMC7022557 31947676

[35] Peng Y, Wang Y, Zhou C, Mei W, Zeng C. PI3K/AKT/mTOR pathway and its role in cancer therapeutics: are we making headway? Front Oncol. 2022;12:819128. 10.3389/fonc.2022.819128 PMC8987494 35402264

[36] Guerau-de-Arellano M, Piedra-Quintero ZL, Tsichlis PN. AKT isoforms in the immune system. Front Immunol. 2022;13:990874. 10.3389/fimmu.2022.990874 PMC9445622 36081513

[37] Jiang N, Dai Q, Su X, Fu J, Feng X, Peng J. Role of PI3K/AKT pathway in cancer: the framework of malignant behavior. Mol Biol Rep. 2020;47(6):4587–629. 10.1007/s11033-020-05435-1 PMC7295848 32333246

[38] Miricescu D, Totan A, Stanescu II S, Badoiu SC, Stefani C, Greabu M. PI3K/AKT/mTOR signaling pathway in breast cancer: from molecular landscape to clinical aspects. Int J Mol Sci. 2020;22(1):173. 10.3390/ijms22010173 PMC7796017 33375317

[39] Rascio F, Spadaccino F, Rocchetti MT, Castellano G, Stallone G, Netti GS, et al. The pathogenic role of PI3K/AKT pathway in cancer onset and drug resistance: an updated review. Cancers. 2021;13(16):3949. 10.3390/cancers13163949 PMC8394096 34439105

[40] Jafari M, Ghadami E, Dadkhah T, Akhavan-Niaki H. PI3K/AKT signaling pathway: erythropoiesis and beyond. J Cell Physiol. 2019;234(3):2373–85. 10.1002/jcp.27262. 30192008

[41] Sun K, Luo J, Guo J, Yao X, Jing X, Guo F. The Pi3k/AKT/mTOR signaling pathway in osteoarthritis: a narrative review. Osteoarthritis Cartilage. 2020;28(4):400–9. 10.1016/j.joca.2020.02.027. 32081707

[42] Zhao B, Yin Q, Fei Y, Zhu J, Qiu Y, Fang W, et al. Research progress of mechanisms for tight junction damage on blood–brain barrier inflammation. Arch Physiol Biochem. 2022;128(6):1579–90. 10.1080/13813455.2020.1784952. 32608276

[43] Zou P, Yang F, Ding Y, Zhang D, Liu Y, Zhang J, et al. Lipopolysaccharide downregulates the expression of zo-1 protein through the akt pathway. BMC Infect Dis. 2022;22(1):774. 10.1186/s12879-022-07752-1 PMC9533599 36199030

[44] Zheng T, Jiang T, Ma H, Zhu Y, Wang M. Targeting PI3K/AKT in cerebral ischemia reperfusion injury alleviation: from signaling networks to targeted therapy. Mol Neurobiol. 2024;61(10):7930–49. 10.1007/s12035-024-04039-1. 38441860

[45] Liu T, Li X, Zhou X, Chen W, Wen A, Liu M, et al. Pi3k/akt signaling and neuroprotection in ischemic stroke: molecular mechanisms and therapeutic perspectives. Neural Regener Res. 2025;20(10):2758–75. 10.4103/NRR.NRR-D-24-00568 PMC11826468 39435629

[46] He X, Li Y, Deng B, Lin A, Zhang G, Ma M, et al. The PI3K/AKT signalling pathway in inflammation, cell death and glial scar formation after traumatic spinal cord injury: mechanisms and therapeutic opportunities. Cell Proliferation. 2022;55(9):e13275. 10.1111/cpr.13275 PMC9436900 35754255

[47] Aliyari M, Ghoflchi S, Hashemy SI, Hashemi SF, Reihani A, Hosseini H. The PI3K/AKT pathway: a target for curcumin’s therapeutic effects. J Diabetes Metab Disord. 2025;24(1):52. 10.1007/s40200-025-01563-2 PMC11748622 39845908

[48] Lee DC, Choi H, Oh JM, Lee J, Lee J, Lee HY, et al. Urban particulate matter regulates tight junction proteins by inducing oxidative stress via the AKT signal pathway in human nasal epithelial cells. Toxicol Lett. 2020;333:33–41. 10.1016/j.toxlet.2020.07.017. 32687961

[49] Zhao S, Chen F, Yin Q, Wang D, Han W, Zhang Y. Reactive oxygen species interact with NLRP3 inflammasomes and are involved in the inflammation of sepsis: from mechanism to treatment of progression. Front Physiol. 2020;11:571810. 10.3389/fphys.2020.571810 PMC7723971 33324236

[50] Rekha A, Afzal M, Babu MA, Menon SV, Nathiya D, Supriya S, et al. Gsk-3β dysregulation in aging: implications for Tau pathology and Alzheimer’s disease progression. Mol Cell Neurosci. 2025;133:104005. 10.1016/j.mcn.2025.104005. 40120784

[51] Liu Q, Telezhkin V, Jiang W, Gu Y, Wang Y, Hong W, et al. Electric field stimulation boosts neuronal differentiation of neural stem cells for spinal cord injury treatment via pi3k/akt/gsk-3β/β-catenin activation. Cell Biosci. 2023;13(1):4. 10.1186/s13578-023-00954-3 PMC9830810 36624495

[52] Zhang Z, Wang L, Li F, Qian X, Hong Z, Wu L, et al. Therapeutic effects of human umbilical cord mesenchymal stem cell on sepsis-associated encephalopathy in mice by regulating pi3k/akt pathway. J Integr Neurosci. 2022;21(1):38. 10.31083/j.jin2101038. 35164474

[53] Yang M, He Y, Xin Y, Jiang J, Tian M, Tan J, et al. Identification of biomarkers and therapeutic targets related to sepsis-associated encephalopathy in rats by quantitative proteomics. BMC Genomics. 2023;24(1):4. 10.1186/s12864-022-09101-7 PMC9814352 36600206

[54] Lin SP, Zhu L, Shi H, Ye S, Li Q, Yin X, et al. Puerarin prevents sepsis-associated encephalopathy by regulating the akt1 pathway in microglia. Phytomedicine. 2023;121:155119. 10.1016/j.phymed.2023.155119. 37801894

[55] Gu M, Mei XL, Zhao YN. Sepsis and cerebral dysfunction: BBB damage, neuroinflammation, oxidative stress, apoptosis and autophagy as key mediators and the potential therapeutic approaches. Neurotox Res. 2021;39(2):489–503. 10.1007/s12640-020-00270-5. 32876918

[56] Yin L, Chen X, Ji H, Gao S. Dexmedetomidine protects against sepsis‑associated encephalopathy through HSP90/AKT signaling. Mol Med Rep. 2019;20(5):4731–40. 10.3892/mmr.2019.10718. 31702043

[57] Kawakami M, Hattori M, Ohashi W, Fujimori T, Hattori K, Takebe M, et al. Role of g protein-coupled receptor kinase 2 in oxidative and nitrosative stress-related neurohistopathological changes in a mouse model of sepsis-associated encephalopathy. J Neurochem. 2018;145(6):474–88. 10.1111/jnc.14329. 29500815

[58] Pan T, Sun S, Chen Y, Tian R, Chen E, Tan R, et al. Immune effects of PI3K/AKT/HIF-1α-regulated glycolysis in polymorphonuclear neutrophils during sepsis. Crit Care. 2022;26(1):29. 10.1186/s13054-022-03893-6 PMC8796568 35090526

[59] Wan P, Tan X, Xiang Y, Tong H, Yu M. PI3K/AKT and cd40l signaling regulate platelet activation and endothelial cell damage in sepsis. Inflammation. 2018;41(5):1815–24. 10.1007/s10753-018-0824-5. 29956071

[60] Zhang L, Li B, Li W, Jiang J, Chen W, Yang H, et al. Mir-107 attenuates sepsis-induced myocardial injury by targeting pten and activating the PI3K/AKT signaling pathway. Cell Tissues Organs. 2023;212(6):523–34. 10.1159/000525476. 35717938

[61] Sun LJ, Qiao W, Xiao YJ, Cui L, Wang X, Ren WD. Naringin mitigates myocardial strain and the inflammatory response in sepsis-induced myocardial dysfunction through regulation of PI3K/AKT/NF-κB pathway. Int Immunopharmacol. 2019;75:105782. 10.1016/j.intimp.2019.105782. 31376623

[62] Hassan D, Menges CW, Testa JR, Bellacosa A. AKT kinases as therapeutic targets. J Exp Clin Cancer Res. 2024;43(1):313. 10.1186/s13046-024-03207-4 PMC11606119 39614261

[63] Wang N, Wang M. Dexmedetomidine suppresses sevoflurane anesthesia-induced neuroinflammation through activation of the PI3K/AKT/mTOR pathway. BMC Anesthesiol. 2019;19(1):134. 10.1186/s12871-019-0808-5 PMC6661092 31351473

[64] Tang G, Yang H, Chen J, Shi M, Ge L, Ge X, et al. Metformin ameliorates sepsis-induced brain injury by inhibiting apoptosis, oxidative stress and neuroinflammation via the PI3K/AKT signaling pathway. Oncotarget. 2017;8(58):97977–89. 10.18632/oncotarget.20105 PMC5716707 29228667

[65] Li L, Jiang W, Yu B, Liang H, Mao S, Hu X, et al. Quercetin improves cerebral ischemia/reperfusion injury by promoting microglia/macrophages m2 polarization via regulating PI3K/AKT/NF-κb signaling pathway. Biomed Pharmacother. 2023;168:115653. 10.1016/j.biopha.2023.115653. 37812891

[66] Tian Y, Liu B, Li Y, Zhang Y, Shao J, Wu P, et al. Activation of rarα receptor attenuates neuroinflammation after sah via promoting m1-to-m2 phenotypic polarization of microglia and regulating MAFB/MSR1/PI3K-AKT/NF-κb pathway. Front Immunol. 2022;13:839796. 10.3389/fimmu.2022.839796 PMC8882645 35237277

[67] Li T, Liu T, Chen X, Li L, Feng M, Zhang Y, et al. Microglia induce the transformation of a1/a2 reactive astrocytes via the CXCR7/PI3K/AKT pathway in chronic post-surgical pain. J Neuroinflammation. 2020;17(1):211. 10.1186/s12974-020-01891-5 PMC7362409 32665021

[68] Chu E, Mychasiuk R, Hibbs ML, Semple BD. Dysregulated phosphoinositide 3-kinase signaling in microglia: shaping chronic neuroinflammation. J Neuroinflammation. 2021;18(1):276. 10.1186/s12974-021-02325-6 PMC8627624 34838047

[69] Yu Y, Shen Q, Lai Y, Park SY, Ou X, Lin D, et al. Anti-inflammatory effects of curcumin in microglial cells. Front Pharmacol. 2018;9:386. 10.3389/fphar.2018.00386 PMC5922181 29731715

[70] Liu B, Zhang Y, Yang Z, Liu M, Zhang C, Zhao Y, et al. Ω-3 dpa protected neurons from neuroinflammation by balancing microglia m1/m2 polarizations through inhibiting Nf-κb/mapk p38 signaling and activating neuron-BDNF-PI3K/AKT pathways. Mar Drugs. 2021;19(11):587. 10.3390/md19110587 PMC8619469 34822458

[71] Saw G, Krishna K, Gupta N, Soong TW, Mallilankaraman K, Sajikumar S, et al. Epigenetic regulation of microglial phosphatidylinositol 3-kinase pathway involved in long-term potentiation and synaptic plasticity in rats. Glia. 2020;68(3):656–69. 10.1002/glia.23748 PMC7003906 31702864

[72] Lu R, Zhang L, Wang H, Li M, Feng W, Zheng X. Echinacoside exerts antidepressant-like effects through enhancing BDNF-CREB pathway and inhibiting neuroinflammation via regulating microglia m1/m2 polarization and jak1/stat3 pathway. Front Pharmacol. 2022;13:993483. 10.3389/fphar.2022.993483 PMC9846169 36686689

[73] Ye ZY, Xing HY, Wang B, Liu M, Lv PY. Dl-3-n-butylphthalide protects the blood–brain barrier against ischemia/hypoxia injury via upregulation of tight junction proteins. Chin Med J. 2019;132(11):1344–53. 10.1097/CM9.0000000000000232 PMC6629356 30939485

[74] Zhao X, Zeng H, Lei L, Tong X, Yang L, Yang Y, et al. Tight junctions and their regulation by non-coding rnas. Int J Biol Sci. 2021;17(3):712–27. 10.7150/ijbs.45885 PMC7975691 33767583

[75] Chen J, Zhang X, Liu X, Zhang C, Shang W, Xue J, et al. Ginsenoside rg1 promotes cerebral angiogenesis via the PI3K/AKT/mTOR signaling pathway in ischemic mice. Eur J Pharmacol. 2019;856:172418. 10.1016/j.ejphar.2019.172418. 31132356

[76] Wang HJ, Ran HF, Yin Y, Xu XG, Jiang BX, Yu SQ, et al. Catalpol improves impaired neurovascular unit in ischemic stroke rats via enhancing VEGF-PI3K/AKT and VEGF-MEK1/2/ERK1/2 signaling. Acta Pharmacol Sin. 2022;43(7):1670–85. 10.1038/s41401-021-00803-4 PMC9253350 34795412

[77] Nan W, He Y, Wang S, Zhang Y. Molecular mechanism of ve-cadherin in regulating endothelial cell behaviour during angiogenesis. Front Physiol. 2023;14:1234104. 10.3389/fphys.2023.1234104 PMC10433914 37601629

[78] Zhi M, Huang J, Jin X. Clinical value of serum neuron-specific enolase in sepsis-associated encephalopathy: A systematic review and meta-analysis. Syst Rev. 2024;13(1):191. 10.1186/s13643-024-02583-4 PMC11265151 39039544

[79] Gong Z, Lao D, Wu Y, Li T, Lv S, Mo X, et al. Inhibiting PI3K/AKT- signaling pathway improves neurobehavior changes in anti-nmdar encephalitis mice by ameliorating blood–brain barrier disruption and neuronal damage. Cell Mol Neurobiol. 2023;43(7):3623–37. 10.1007/s10571-023-01371-3 PMC10477152 37314618

[80] Zhang Q, Yao M, Qi J, Song R, Wang L, Li J, et al. Puerarin inhibited oxidative stress and alleviated cerebral ischemia-reperfusion injury through PI3K/AKT/NRF2 signaling pathway. Front Pharmacol. 2023;14:1134380. 10.3389/fphar.2023.1134380 PMC10240043 37284311

[81] Fedder-Semmes KN, Appel B. The AKT-mTOR pathway drives myelin sheath growth by regulating cap-dependent translation. J Neurosci. 2021;41(41):8532–44. 10.1523/JNEUROSCI.0783-21.2021 PMC8513705 34475201

[82] Yoshii A, Constantine-Paton M. Postsynaptic localization of psd-95 is regulated by all three pathways downstream of trkb signaling. Front Synaptic Neurosci. 2014;6:6. 10.3389/fnsyn.2014.00006 PMC3978359 24744726

[83] Udoh UG, Bruno JR, Osborn PO, Pratt KG. Serotonin strengthens a developing glutamatergic synapse through a pi3k-dependent mechanism. J Neurosci. 2024;44(6):e1260232023. 10.1523/JNEUROSCI.1260-23.2023 PMC10860612 38169457

[84] Gong GQ, Bilanges B, Allsop B, Masson GR, Roberton V, Askwith T, et al. A small-molecule pi3kα activator for cardioprotection and neuroregeneration. Nature. 2023;618(7963):159–68. 10.1038/s41586-023-05972-2 PMC7614683 37225977

[85] Wallroth A, Koch PA, Marat AL, Krause E, Haucke V. Protein kinase n controls a lysosomal lipid switch to facilitate nutrient signalling via mtorc1. Nat Cell Biol. 2019;21(9):1093–101. 10.1038/s41556-019-0377-3. 31451768

[86] Wang L, Zhang L. Protein kinase N2 reduces hydrogen peroxide-induced damage and apoptosis in pc12 cells by antioxidative stress and activation of the mtor pathway. Evid Based Complement Alternat Med. 2022;2022:2483669. 10.1155/2022/2483669 PMC9519335 36185087

[87] Baffi TR, Lordén G, Wozniak JM, Feichtner A, Yeung W, Kornev AP, et al. Mtorc2 controls the activity of PKC and AKT by phosphorylating a conserved tor interaction motif. Sci Signal. 2021;14(678):eabe4509. 10.1126/scisignal.abe4509 PMC8208635 33850054

[88] Gao Q, Hernandes MS. Sepsis-associated encephalopathy and blood–brain barrier dysfunction. Inflammation. 2021;44(6):2143–50. 10.1007/s10753-021-01501-3 PMC11044530 34291398

[89] Bai Y, Li L, Dong B, Ma W, Chen H, Yu Y. Phosphorylation-mediated pi3k-art signalling pathway as a therapeutic mechanism in the hydrogen-induced alleviation of brain injury in septic mice. J Cell Mol Med. 2022;26(22):5713–27. 10.1111/jcmm.17568 PMC9667523 36308410

[90] Liu A, Zhu Y, Chen W, Merlino G, Yu Y. Pten dual lipid- and protein-phosphatase function in tumor progression. Cancers (Basel). 2022;14(15):3666. 10.3390/cancers14153666 PMC9367293 35954330

[91] Li C, Yu T, Li W, Gong L, Shi J, Liu H, et al. Pink1 deficiency with Ca(2+) changes in the hippocampus exacerbates septic encephalopathy in mice. Chem Biol Interact. 2023;374:110413. 10.1016/j.cbi.2023.110413. 36804394

[92] Liu L, Zhang J, Han Y, Liu D. The mechanism of girdin in degenerative brain disease caused by high glucose stimulation. Front Endocrinol (Lausanne). 2022;13:892897. 10.3389/fendo.2022.892897 PMC9623676 36329890

[93] Enomoto A, Ping J. Takahashi MGirdin, a novel actin-binding protein, and its family of proteins possess versatile functions in the akt and wnt signaling pathways. Ann N Y Acad Sci. 2006;1086:169–84. 10.1196/annals.1377.016. 17185515

[94] Hu JT, Li Y, Yu B, Gao GJ, Zhou T, Li S. Girdin/giv is upregulated by cyclic tension, propagates mechanical signal transduction, and is required for the cellular proliferation and migration of mg-63 cells. Biochem Biophys Res Commun. 2015;464(2):493–9. 10.1016/j.bbrc.2015.06.165. 26163263

[95] Cao M, Tan X, Jin W, Zheng H, Xu W, Rui Y, et al. Upregulation of ras homolog enriched in the brain (rheb) in lipopolysaccharide-induced neuroinflammation. Neurochem Int. 2013;62(4):406–17. 10.1016/j.neuint.2013.01.025. 23391520

[96] Deng L, Chen L, Zhao L, Xu Y, Peng X, Wang X, et al. Ubiquitination of rheb governs growth factor-induced mtorc1 activation. Cell Res. 2019;29(2):136–50. 10.1038/s41422-018-0120-9 PMC6355928 30514904

[97] Jiang J, Zhang L, Zou J, Liu J, Yang J, Jiang Q, et al. Phosphorylated s6k1 and 4e-bp1 play different roles in constitutively active rheb-mediated retinal ganglion cell survival and axon regeneration after optic nerve injury. Neural Regener Res. 2023;18(11):2526–34. 10.4103/1673-5374.371372 PMC10360084 37282486

[98] Brenner CM, Choudhary M, McCormick MG, Cheung D, Landesberg GP, Wang JF, et al. Bag3: nature’s quintessential multi-functional protein functions as a ubiquitous intra-cellular glue. Cells. 2023;12(6):937. 10.3390/cells12060937 PMC10047307 36980278

[99] Ashare A, Nymon AB, Doerschug KC, Morrison JM, Monick MM, Hunninghake GW. Insulin-like growth factor-1 improves survival in sepsis via enhanced hepatic bacterial clearance. Am J Respir Crit Care Med. 2008;178(2):149–57. 10.1164/rccm.200709-1400OC PMC2453509 18436791

[100] Xu L, Zhang W, Sun R, Liu J, Hong J, Li Q, et al. Igf-1 may predict the severity and outcome of patients with sepsis and be associated with microrna-1 level changes. Exp Ther Med. 2017;14(1):797–804. 10.3892/etm.2017.4553 PMC5488740 28673002

[101] Jin W. Regulation of BDNF-TRKB signaling and potential therapeutic strategies for parkinson’s disease. J Clin Med. 2020;9(1):257. 10.3390/jcm9010257 PMC7019526 31963575

[102] Hua Z, Gu X, Dong Y, Tan F, Liu Z, Thiele CJ, et al. PI3K and MAPK pathways mediate the BDNF/TRKB-increased metastasis in neuroblastoma. Tumour Biol. 2016;37(12):16227–36. 10.1007/s13277-016-5433-z PMC5250655 27752996

[103] Hou D, Liao H, Hao S, Liu R, Huang H, Duan C. Curcumin simultaneously improves mitochondrial dynamics and myocardial cell bioenergy after sepsis via the sirt1-drp1/pgc-1α pathway. Heliyon. 2024;10(7):e28501. 10.1016/j.heliyon.2024.e28501 PMC10998060 38586339

[104] Owjfard M, Rahimian Z, Karimi F, Borhani-Haghighi A, Mallahzadeh A. A comprehensive review on the neuroprotective potential of resveratrol in ischemic stroke. Heliyon. 2024;10(14):e34121. 10.1016/j.heliyon.2024.e34121 PMC11284444 39082038

[105] Sun P, Yang Y, Yang L, Qian Y, Liang M, Chen H, et al. Quercetin protects blood–brain barrier integrity via the PI3K/AKT/ERK signaling pathway in a mouse model of meningitis induced by glaesserella parasuis. Biomolecules. 2024;14(6):696. 10.3390/biom14060696 PMC11201931 38927100

[106] Zhang B, Wang Y, Li H, Xiong R, Zhao Z, Chu X, et al. Neuroprotective effects of salidroside through PI3K/AKT pathway activation in Alzheimer’s disease models. Drug Des Dev Ther. 2016;10:1335–43. 10.2147/DDDT.S99958 PMC4827895 27103787

[107] Sharma R, Mehan S, Khan Z, Das Gupta G, Narula AS. Therapeutic potential of oleanolic acid in modulation of pi3k/akt/mtor/stat-3/gsk-3β signaling pathways and neuroprotection against methylmercury-induced neurodegeneration. Neurochem Int. 2024;180:105876. 10.1016/j.neuint.2024.105876. 39368746

[108] Ren ZX, Zhao YF, Cao T, Zhen XC. Dihydromyricetin protects neurons in an mptp-induced model of Parkinson’s disease by suppressing glycogen synthase kinase-3 beta activity. Acta Pharmacol Sin. 2016;37(10):1315–24. 10.1038/aps.2016.42 PMC5057232 27374489

[109] Xiao CL, Yin WC, Zhong YC, Luo JQ, Liu LL, Liu WY, et al. The role of PI3K/AKT signalling pathway in spinal cord injury. Biomed Pharmacother. 2022;156:113881. 10.1016/j.biopha.2022.113881. 36272264

[110] Khezri MR, Jafari R, Yousefi K, Zolbanin NM. The PI3K/AKT signaling pathway in cancer: molecular mechanisms and possible therapeutic interventions. Exp Mol Pathol. 2022;127:104787. 10.1016/j.yexmp.2022.104787. 35644245

[111] He Y, Sun MM, Zhang GG, Yang J, Chen KS, Xu WW, et al. Targeting PI3K/AKT signal transduction for cancer therapy. Signal Transduct Target Ther. 2021;6(1):425. 10.1038/s41392-021-00828-5 PMC8677728 34916492

[112] Bird ST, Tian F, Flowers N, Przepiorka D, Wang R, Jung TH, et al. Idelalisib for treatment of relapsed follicular lymphoma and chronic lymphocytic leukemia: a comparison of treatment outcomes in clinical trial participants vs medicare beneficiaries. JAMA Oncol. 2020;6(2):248–54. 10.1001/jamaoncol.2019.3994 PMC6990831 31855259

[113] Blair HA. Duvelisib: first global approval. Drugs. 2018;78(17):1847–53. 10.1007/s40265-018-1013-4. 30430368

[114] Rusquec P, Blonz C, Frenel JS, Campone M. Targeting the PI3K/AKT/mTOR pathway in estrogen-receptor positive her2 negative advanced breast cancer. Ther Adv Med Oncol. 2020;12:1758835920940939. 10.1177/1758835920940939 PMC7388095 32782489

[115] Hedayati N, Safari MH, Milasi YE, Kahkesh S, Farahani N, Khoshnazar SM, et al. Modulation of the PI3K/AKT signaling pathway by resveratrol in cancer: molecular mechanisms and therapeutic opportunity. Discover Oncol. 2025;16(1):669. 10.1007/s12672-025-02471-w PMC12052642 40323335

[116] Bai Y, Mi W, Meng X, Dong B, Jiang Y, Lu Y, et al. Hydrogen alleviated cognitive impairment and blood‒brain barrier damage in sepsis-associated encephalopathy by regulating abc efflux transporters in a pparα-dependent manner. BMC Neurosci. 2023;24(1):37. 10.1186/s12868-023-00795-3 PMC10360271 37474902

[117] Chen JY. Research progress on the mechanisms of resveratrol in the prevention and treatment of cerebral ischemia-reperfusion injury. Adv Clin Med. 2024;14(3):228–33.

[118] Wright B, King S, Suphioglu C. The importance of phosphoinositide 3-kinase in neuroinflammation. Int J Mol Sci. 2024;25(21):11638. 10.3390/ijms252111638 PMC11546674 39519189

[119] Fakhri S, Iranpanah A, Gravandi MM, Moradi SZ, Ranjbari M, Majnooni MB, et al. Natural products attenuate PI3K/AKT/mTOR signaling pathway: a promising strategy in regulating neurodegeneration. Phytomedicine. 2021;91:153664. 10.1016/j.phymed.2021.153664. 34391082

[120] Wiegman CH, Li F, Ryffel B, Togbe D, Chung KF. Oxidative stress in ozone-induced chronic lung inflammation and emphysema: a facet of chronic obstructive pulmonary disease. Front Immunol. 2020;11:1957. 10.3389/fimmu.2020.01957 PMC7492639 32983127

